# Competition in California’s Medi-Cal Managed Care Market Assessed by Herfindahl-Hirschman Index

**DOI:** 10.1177/00469580221127063

**Published:** 2022-09-27

**Authors:** Michael Tawil, Anthony M. DiGiorgio

**Affiliations:** 1 School of Medicine, University of California, San Francisco, CA, USA; 2 Department of Neurological Surgery, University of California, San Francisco, CA, USA; 3 Institute for Health Policy Studies, University of California, San Francisco, CA, USA

**Keywords:** Medi-Cal, Medicaid, managed care, insurance competition, Herfindahl-Hirschman Index, healthcare economics

## Abstract

Evaluating market competition is an important practice to assess how the forces and components at play in a select market interact. Healthcare markets are similar to any other market present in the world, where competition can be present or absent in the exchange of goods and services. Applying a standard measure of assessing market competition, the Herfindahl-Hirschman Index, to California’s Medi-Cal managed care marketplace, it is found that there is no competition present in all of California’s counties as defined by the common interpretation of the Herfindahl-Hirschman Index. A distinctive trend in markets is that when less competition is present, the cost of goods and services increases to reflect the principles of supply and demand. California Medi-Cal markets follow this trend of less competitive markets being associated with increased adult midpoint costs. These findings help further to elucidate California’s Medi-Cal marketplace on a county-by county level.

**What do we already know about this topic?**

Insurance marketplaces have varying degrees of competition and patient choice but there are few studies on competition within Medicaid Managed Care marketplaces.

**How does your research contribute to the field?**

California employs a unique county-based Medicaid Managed Care system. This study provides a novel snapshot of California’s Medi-Cal managed-care marketplace by implementing the Herfindahl-Hirschman Index to assess the competition on a county level and its association with adult midpoint costs.

**What are your research’s implications toward theory, practice, or policy?**

The analysis completed as part of this study provides a current overview of competition in California Medi-Cal marketplaces which will help inform the public and policymakers of Medi-Cal’s standing, and it provides an important reference point to determine the impact of alterations to California’s Medi-Cal landscape.

## Introduction

California’s Medicaid program, dubbed Medi-Cal, is the largest Medicaid program in the nation.^1^ It was the first program to pilot managed care in Medicaid and has been turning to a near-exclusive use of the Managed Care Organization (MCO) model ever since.^1^ MCO plans in California are largely regulated by the state’s Knox-Keene Act. Medi-Cal also uses a county-based administrative system, with different MCO structures in differing counties. There are 4 basic county-based MCO structures in this system: County Organized Health Systems (COHS), 2-Plan, Regional, and Geographic Managed Care (GMC) (Table 1).^2^ This allows for varying levels of competition and patient choice between Medi-Cal MCOs among neighboring counties.

**Table 1.** California Medi-Cal Managed Care Organization Models.

MCO Model	COHS	GMC	2 plan	Regional	Imperial	San Benito
Description	County run MCO model. This is the only managed care plan available in these counties.	This MCO model contracts with multiple commercial health plans. All contracted health plans have to go through the DHCS procurement process.	Counties with this MCO have a county-organized plan and a commercial plan. Commercial plans are required to go through the DHCS procurement process.	This MCO plan consists of 2 commercial plans. Commercial plans that are interested are required to go through the DHCS procurement process.	This plan came about from the Regional MCO model. This plan also consists of 2 commercial plans that are required to go through the DHCS procurement process.	This MCO model stems from the Regional MCO model. There is only one commercial plan available that is required to go through the DHCS procurement process. Beneficiaries have to option to select this managed-care plan or fee-for-service Medi-Cal.
Knox-Keene (Y/N)	N	Y	Y	Y	Y	Y

*Source.* (Adapted from DHCS).^2^

Competition is a way to ensure Americans have choices between high-quality health care plans.^3^ Despite the variability in coverage options, however, patients often lack choice between methods of financing their healthcare. While there are various public-private partnerships in both Medicare and Medicaid, even these options leave limited choices for patients. A recent study by the American Medical Association assessed the competition present in the United States metropolitan statistical areas (MSA). They found that the mean Herfindahl-Hirschman Index (HHI) (the standard economic measure for market concentration) across Preferred Provider Organization (PPO), Health Maintenance Organization (HMO), Point of Service (POS), and exchange marketplaces are highly concentrated with mean HHIs of 4326, 5702, 7892, and 6240 respectively.^4^

Within Medicaid, patients likely face even fewer choices. Medicaid programs in the United States are managed on the state level. Due to this, each state’s Medicaid program is unique in coverages and plans offered to their constituents. Like Medicare, Medicaid offers either fee for service (FFS) or MCO models. In the MCO model, private groups received a risk-adjusted capitated rate for managing enrollees.

Each of the California county based MCO models are distinct. COHS plans are run by the county government and are the only plan available to Medi-Cal recipients in the specific county. These plans are instituted and overseen by a commission appointed by the county board of supervisors. Two-plan models are composed of a local county-run plan and a single commercial plan, both with Knox-Keene licenses. The 2-plan models cover most Medi-Cal participants with 6.5 million enrollees (64%) as of 2015.^1^ In 2-plan counties, the county-run plans are instituted by county ordinance while commercial plans must go through the DHCS procurement process. Regional models offer 2 commercial plans that must be Knox-Keene licensed and go through the DHCS procurement process. GMC plans are composed of more than 2 commercial health plans. They must go through the DHCS procurement process and meet the specific requirements of Medi-Cal managed care plans as well (Table 1).

The purpose of our study is to examine the California Medicaid MCO marketplace through a lens of market concentration and patient choice.

## Methods

California Medi-Cal managed care plan data was extracted from the Department of Health Care Services online database that is publicly accessible.^5^ Competition in managed-care marketplaces was calculated using the Herfindahl-Hirschman Index (HHI), a standard model for assessing competition. This was done for each county. Its usage in this context is supported by the Department of Justice and Federal Trade Commission.^6,7^

To calculate HHI for each county, we used the Medi-Cal enrollment database of total eligible per county, further subdivided into the number of eligible for each health plan offered in the county. We excluded fee-for-service and out-of-county health plans from our HHI calculation as they are not managed health care plans encompassed under Medi-Cal. HHI per county was calculated by squaring each managed-care healthcare plan’s market percentage and taking the total sum.^6^ The possible range of HHI is between zero for a very competitive market to 10 000, indicating a very concentrated market with only one seller of a good or service. The DOJ classifies a market with minimal concentration with an HHI of 0 to 1500, moderately concentrated from 1500 to 2500, and highly concentrated as greater than 2500.^6^

To analyze how competition correlates with cost, we specifically utilized Medi-Cal’s financial reports for each managed care plan offered in California. This included COHS, GMC, 2-Plan, and Regional Plan data reported per county in each fiscal year. We analyzed financial reports from the 2018-19 fiscal year for each managed care plan. These give the capitated rate paid to each MCO per enrollee. Adult midpoint costs (AMC) for health care plan rates were the targeted metric in our study. Calculating the weighted average of adult midpoint rates utilized the total eligible enrolled in each county managed plan combined with AMC for each plan in the county to achieve the most representative Medi-Cal adult midpoint rate per county.

County HHI and weighted average of AMC data points were then utilized to calculate the Pearson Product-Moment correlation coefficient to determine if any correlation existed between these 2 variables. Average HHI and average AMC were also used via 1-way ANOVA to compare the different county MCO types, with San Benito being combined with COHS counties and Imperial combined with regional counties.

## Results

**Figure 1.**
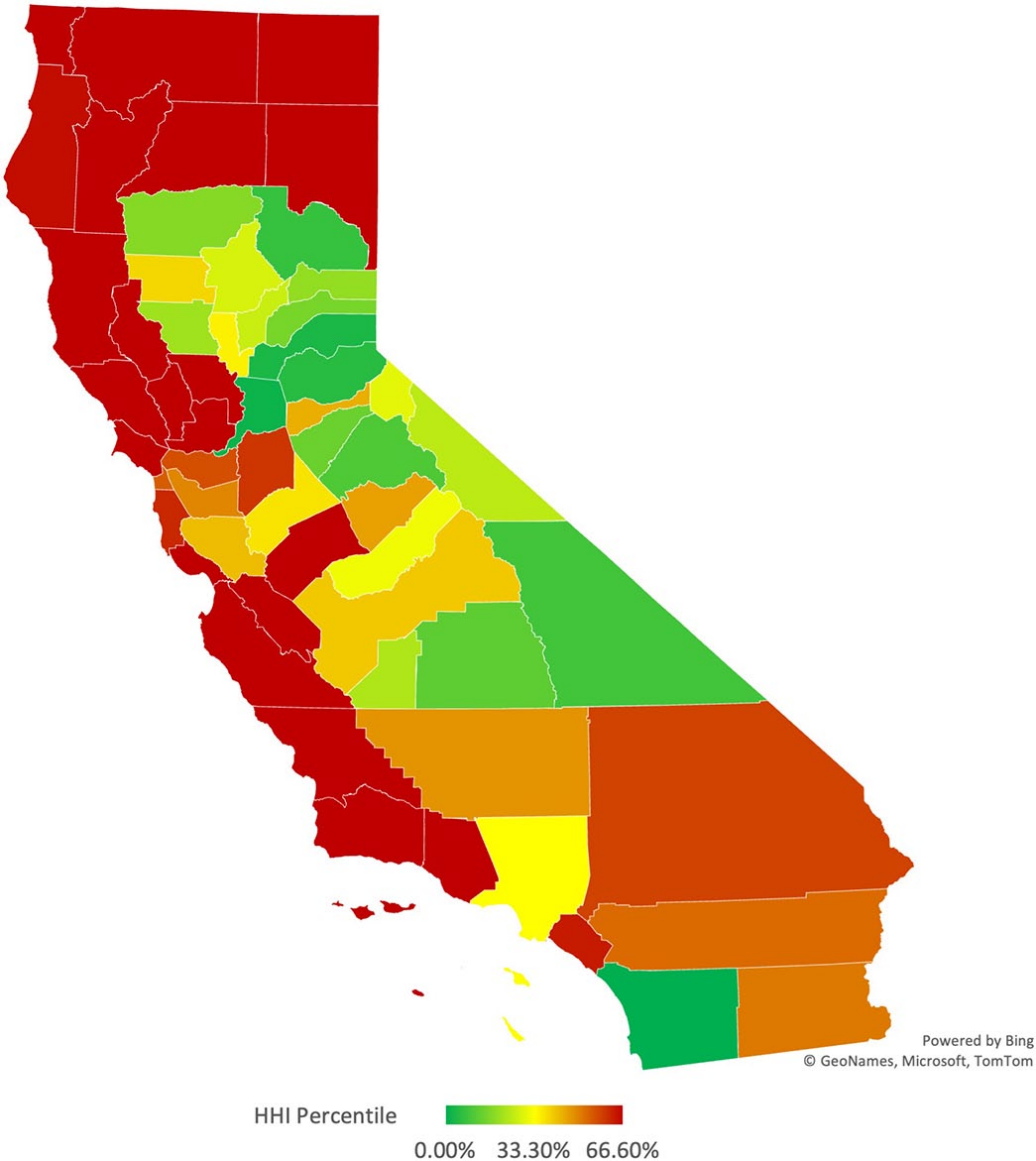
California Medi-Cal Managed Care plans Herfindahl-Hirschman Index—Heat map by County.

We calculated the Herfindahl-Hirschman Index for each county using the above methods (Figure 1). We found that every California county’s Medi-Cal managed-care plan is highly concentrated, with an HHI greater than 2500. In the upper range, it was calculated that 20 of the 58 counties in California had the maximum HHI possible of 10 000 (a single MCO provider). The interquartile range for all countries was 4701, with the 25th percentile being 5299 and the 75th percentile being 10 000. The most competitive insurance markets in California are the counties of Sacramento and San Diego, with HHI’s of 2880 and 2535, respectively. Notably, these are the 2 GMC plan counties.

**Figure 2.**
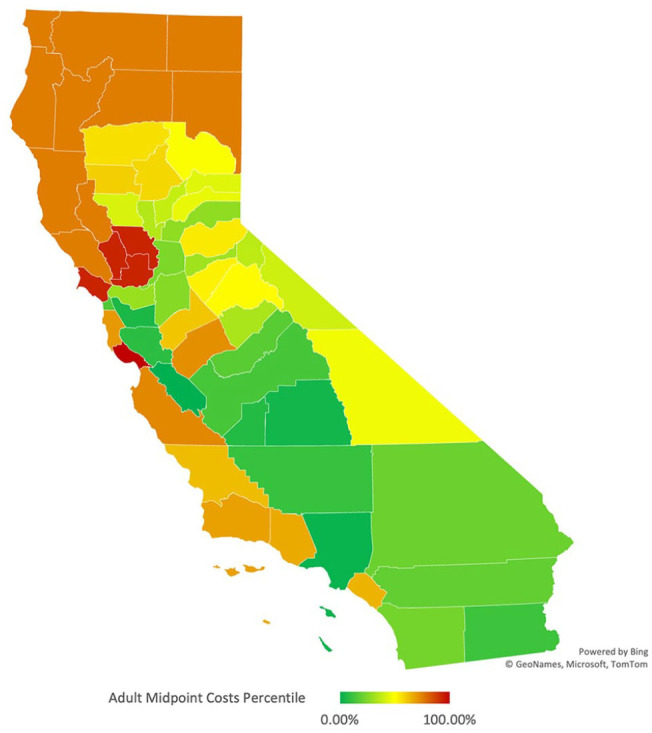
California Medi-Cal adult midpoint costs—Heat map by County.

Adult midpoint rates per county had more variation (Figure 2). We found the median to be 272 USD, the interquartile range for county rates to be 104 USD, with the 25th percentile being 256 USD and the 75th percentile being 360 USD. Los Angeles and San Benito had the lowest rates of all plans, each having a rate under 200 USD. Fifteen counties had rates over the 75th percentile, with the most expensive county for rates being Santa Cruz at 376 USD.

When comparing average HHI of the county plan types using ANOVA, all 4 (COHS, 2-plan, regional, and GMC) significantly differed from one another using Tukey’s post-hoc analysis. On average AMC, COHS was significantly higher than the other 3 plan types, while the other 3 did not significantly differ from one another (Table 2).

**Table 2.** Average HHI and AMC by County MCO Type.

County type	Average HHI	Average AMC
COHS	9737.4*	345.4*
2-plan	6646.6*	236.5
GMC	2707.4*	254.0
Regional	5425.6*	268.7
Total	7389.7	292.7

* indicates significant difference on ANOVA. All 4 plan types (COHS, 2-plan, regional, and GMC) significantly differed from one another using Tukey’s post-hoc analysis. On average AMC, COHS was significantly higher than the other 3 plan types, while the other 3 did not significantly differ from one another.

We utilized the Pearson Product-Moment correlation coefficient to determine the correlation between these 2 variables, county HHI and adult midpoint rates. A positive correlation was found of +.73. This correlation can be visualized in California heat maps, whereas when HHI increases in more concentrated marketplaces, adult midpoint premiums accompany this increase (Figures 1 and 2).

## Discussion

While California, in total, has more Medicaid MCO plans than any other state, because of the design on a county level, patients still largely lack choice. Competition between insurance plans is scarce. Not 1 of the 58 counties in California had a competitive insurance marketplace for Medi-Cal managed care plans as defined by the Department of Justice and Federal Trade Commission. This deprives California MediCal patients of choice and the freedom to move between insurance providers. This leaves them little recourse if they are unsatisfied with their healthcare.

There is not universal agreement that choice and competition are good for Medi-Cal patients, however. Millett et al^8^ examined the California Medi-Cal market, arguing that increase competition led to delays in health plan enrollment and poorer health outcomes. The Medi-Cal MCO landscape has continued to evolve since the time that paper was published. Their methods are also open to criticism. They simply dichotomized patients into counties with and without choices of more than one plan. They did not use a granular measure of competition like HHI. Many of their findings regarding utilization of care are natural byproduct of consumer choice, such as needing time to choose and enroll in a plan. This could be improved with administrative reforms. Their health outcomes are likely multifactorial and are not convincingly tied to patient choice. Our study didn’t investigate any individual health outcomes, but we hope the competition and price data derived here can spur future research.

Without competition, patients must rely on the government to hold MCO plans accountable. Recent investigations started by the Department of Managed Care (DMHC) and the California Department of Health Care Services (DHCS) into LA Care, a managed care plan for Medi-Cal beneficiaries in Los Angeles County, found a notable absence of response to grievances and several delays in approving authorizations.^9^ There are also problems with network adequacy in Medicaid, nationally.^10,11^ California maintains its own network adequacy rules for Medi-Cal MCO plans and issues annual reports on the DHCS website.^12^ “Secret shopper” studies in California have shown inconsistencies in ACA marketplace networks.^13^ This hasn’t been conducted with Medi-Cal, so further research is needed to determine if Medicaid MCO competition affects network adequacy and patient grievances.

Recently, Kaiser Health, which offers their Kaiser Foundation Health plan to GMC and Regional Rural beneficiaries has been approved to expand Medi-Cal coverage state-wide. This ruling is controversial. With this approval Kaiser can enroll beneficiaries without bidding against other Medi-Cal plans, increasing Kaiser’s Medi-Cal enrollment by an estimated 25% throughout 32 California counties.^14^

This Kaiser’s contract will offer an additional option for beneficiaries in counties with limited options. Provisions for “actuarially sound” payments attempt to alleviate concerns about Kaiser cherry-picking sick patients, leaving established MCO plans with more costly beneficiaries.^15^ Having an additional state-wide MCO in the marketplace should increase patient choice and competition. We predict HHI in many counties will improve once the Kaiser plan is implemented in 2024 and we hope this research can serve as a baseline for comparison.

**Figure 3.**
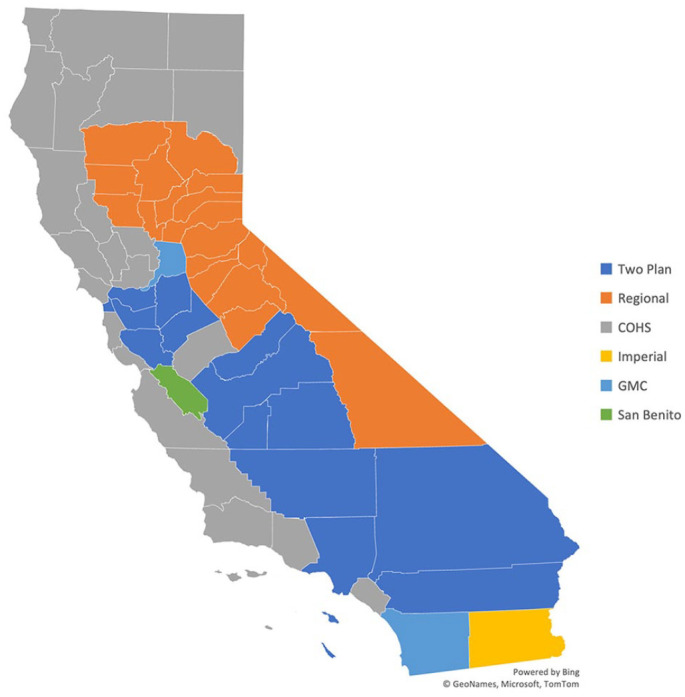
California Medi-Cal Managed Care plans by County (Adapted from DHCS).^16^

Regarding specific plan models, COHS and San Benito plans are a unique subset of California’s Medi-Cal MCO system. All COHS counties in California have HHIs of 10 000, except for Humboldt, Orange, and San Mateo counties (due to some other coordinated care plans). San Benito isn’t technically a COHS county, as their single plan is evolved from and is managed by a commercial entity.^2^ By their vary design, though, these counties have no competition or choice for patients. These COHS counties also have some of California’s most expensive adult midpoint premiums (Figures 2 and 3 and Table 2).

Historically, California was the first state to offer a managed care alternative to fee-for service Medicaid.^1^ Contra Costa County was the first county in California to start a managed care model under Medi-Cal for its beneficiaries. From this initial pilot, Santa Barbara County pioneered the COHS MCO model in 1983, and by the 1990s, the DHCS expanded the managed care COHS model to an additional 17 counties.^17,18^ Alongside this expansion, 2-plan and GMC models appeared in the 1990s. The GMC was the direct result of an Assembly and Senate Bill in California that established this new managed care model specifically in Sacramento and San Diego counties, with the ultimate goal of reducing healthcare costs and increasing beneficiary access to medical care.^19^

The expansion of managed care in California occurred systematically, with more plans becoming available as the state phased out FFS. Counties have the option to change their MCO model. A county can complete a transition to a different managed care plan during DHCS managed care plan procurement periods which has recently started in 2021 and will become effective at the start of 2024.^20^ The history and overall operation of managed care in California demonstrates that much of the limitations in choice in California’s Medi-Cal marketplace are driven at the county level and beneficiary choice depends on their county of residence (as some counties offer more choice than others). However, several federal and state regulations (such as mandates on eligibility and coverages) also restrict competition and choice.

In counties where beneficiaries have multiple plan options under Medi-Cal, they must indicate into which plan they would like to enroll. Beneficiaries have 30 days to choose their plan or they are assigned to a plan.^21^ The DHCS has online resources and a dedicated website that helps beneficiaries compare plans and receive assistance if needed.^22^ Counties also have varying supports for beneficiaries. San Francisco County, for example, provides aid to beneficiaries when signing up under a Medi-Cal plan and continues to assist beneficiaries in maintaining coverage.^23^

When undergoing assignment, enrollees are assigned to a plan if they had prior enrollment or if they have family members in that plan. If that is not the case, they are assigned using an algorithm that favors plans with better performance metrics. Approximately 50% of enrollees are assigned.^15,24^

Medi-Cal beneficiaries can change plans if they wish to do so by phone, in person, or by mailing a completed enrollment choice form for their specific county.^22^ The statewide switch rate is 4.88%. In GMC counties, such as Sacramento and San Diego, the switch rate is 7.79%.^19^ Indicating that beneficiaries take advantage of the increased choice in plans. These counties have Medi-Cal enrollments of 37.3% and 27.6%, respectively (compared to California at 34.5%).^25^ The amount of competition could be the result of an enticing market for insurers as well. The average age of Sacramento is 34.9, San Diego is 35.2, and California is 36.7.^26^

Counties decide which managed care plans to provide to their population. There are advocates for both more and less choice among MCO plans. Proponents for single-plan counties argue that the regulators can craft a plan uniquely designed to cover their citizens and that choice only adds inefficiency.^18,27^ Arguments in favor of more choice are that the bidding and competition will improve both cost and quality, leaving the patient to decide what is best for their own healthcare needs.^27^ While GMC counties have shown slightly worse outcomes in HEDIS performance metrics, beneficiaries appear to benefit from the competition, evidenced by both the higher switch rate and the lower cost.^19^

It is argued that less competition in healthcare insurance markets, measured using the HHI metric, results in higher premiums.^28,29,30^ Our results from this study show these trends persist in a public, capitated Medicaid MCO market. Payers must calculate the average contracted rate (ACR) in a specific method stipulated by Knox-Keene regulations.^31^ This involves calculating the weighted average using the number of claims per different contracts utilized to calculate the base ACR rate for a specific healthcare service code. Counties with COHS plans only must abide by the DHCS contract to provide sufficient coverage and service to insurees.

The monthly capitated rates are based on the prior year’s medical and administrative cost, adjusted for anticipated changes in costs. There are additional adjustments and supplemental rates for changing services and unexpected costs.^32^ Given this calculation, the rates are reflective of cost-management techniques and provides perverse incentive against cutting costs. An MCO that lowers costs is given a lower capitation rate the next year. Also, given that rates are directly correlated with spending, counties with higher poverty rates are likely going to have higher spending and higher rates. The link between poverty & healthcare spending is well established.^33,34^

Polyakova et al^35^ completed a study on Affordable Care Act Health Insurance Marketplaces to assess if any connection was present between hospital, physician, and insurer concentration on healthcare premiums. They found that increased hospital and physician prices were associated with less competitive insurance marketplaces and higher premiums. Due to these findings, they propose that the increased costs of hospitals and physicians, driven by increased consolidation, increase insurance rates which are then passed to insurees in the form of increased premiums.^35^ Also using HHI, Scheffler et al^36^ specifically studied the effect of hospital concentration, medical group concentration, and health plan concentration on premium growth year-over-year from 2014-2015 in California and New York managed care marketplaces for ACA marketplace plans. Significant findings included a positive correlation between hospital concentration and premium growth in both states; however, they found a negative correlation in only California when associating the effect of health plan concentration on premium growth.^36^ Both studies attribute health premium increases to potential forces outside of insurance concentration. Further studies will be required to determine if the correlation we found in 2019 Medi-Cal managed care plans was solely attributed to insurance concentration or a possible supplementary correlation to hospital and medical group concentration.

DHCS has plans to alter and improve California’s Medi-Cal marketplace. One of these changes is the California Advancing and Innovating Medi-Cal (CalAIM) plan. Under this plan, healthcare for Medi-Cal beneficiaries will include additional avenues of support outside traditional healthcare. This extra care will target improving homelessness, behavioral healthcare, vulnerable children, individuals with increased health needs, justice-involved patients, and the aging population in California.^37^ In addition, this plan aims to standardize enrollment and benefits statewide, implement regional pricing, increase behavioral healthcare, add dental benefits, and improve oversight and monitoring on a county level.^37^ It is unclear what the recent Kaiser and CalAIM proposals will ultimately do for patient choice within Medi-Cal.

## Limitations

We utilized Medi-Cal 2018-2019 data as this data set was complete for all metrics of our study, but our findings may not translate to more recent years. Our study will need to be revisited to assess if the positive correlation between HHI and premiums are maintained in Medi-Cal managed care marketplaces. Subsequently, we only utilized adult midpoint premium cost data in our analysis, and our findings may not translate to the lower or upper bound in adult health care premiums. Lastly, some counties have smaller, specialized health plans offered alongside plans by insurers with a larger market cap. DHCS data does not report adult midpoint costs data for these plans, and although having this data would make a small impact on our data, it could alter our findings and is relevant to mention. Of course, California is a very large and diverse state, county-by-county. Some settings will almost certainly benefit from more competition (such as large urban counties). Very large, diverse populations such as those found in the GMC counties like Los Angeles, San Francisco, or Alameda would almost certainly benefit from improved choice, but the evidence for this is more theoretical than empirical.

## Conclusion

Using the Herfindal-Hirschman Index in the setting of Medi-Cal coverage in California, we found that California is highly concentrated with little to no competition in Medi-Cal managed-care marketplaces. Increased consolidation of managed care plans in California positively correlates with increased adult premiums passed along to county residents who rely on Medi-Caid for health coverage. Fewer options in county managed-care coverage impacts consumers’ power of choice when obtaining health coverage.
